# Nutri Veggie Index: An Innovative Nutrition Classification Scheme for Quality Assessment of Plant-Based Meat Analogues

**DOI:** 10.3390/foods15193416

**Published:** 2026-09-24

**Authors:** Nathália Tarossi Locatelli, Sarah Polezi, Mariana Frazão Batista, Daniel Henrique Bandoni, Veridiana Vera de Rosso

**Affiliations:** 1 Food Labeling Observatory, Nutrition and Food Service Research Center (CPPNAC), Federal University of São Paulo (UNIFESP), Santos 11015-020, SP, Brazil; nathalia.locatelli@unifesp.br (N.T.L.); mfbatista@unifesp.br (M.F.B.);; 2 Graduate Program in Nutrition, Federal University of São Paulo (UNIFESP), Santos 11015-020, SP, Brazil

**Keywords:** plant-based meat analogues, nutritional quality, food labeling, nutrition classification schemes

## Abstract

Growing availability and consumption of plant-based meat analogues (PBMAs) have highlighted the need for nutritional assessment tools specifically designed to address the unique characteristics of these products. This study aimed to develop a nutritional quality assessment tool for PBMAs and to evaluate its applicability, agreement with existing nutritional classification schemes, and usability among nutrition professionals. The Nutritional Quality Index for Plant-Based Meat Analogues (NCS–Nutri Veggie Index) was developed based on expert consensus recommendations and existing nutritional assessment frameworks. The index evaluates seven domains: front-of-package nutrition labeling, characteristics associated with protein quality and quantity, lipid composition, sodium content, dietary fiber, micronutrient fortification, and the inclusion of sociobiodiversity ingredients, generating scores ranging from 0 to 100 and classifying products from A (excellent) to E (poor nutritional quality). NCS–Nutri Veggie Index was applied to 334 PBMA. Among the products evaluated, most foods had intermediate nutritional quality (C), corresponding to 53.0% (*n* = 177), followed by good quality (B), 33.5% (*n* = 112). NCS–Nutri Veggie Index showed substantial or excellent agreement with Nutri-Score and the Pan American Health Organization Nutrient Profile Model. The usability questionnaire demonstrated acceptable internal consistency across all evaluated domains. These findings indicate that the tool developed for these products is capable of indicating their nutritional quality.

## 1. Introduction

In recent years, changes in dietary patterns and increasing concerns related to environmental sustainability, human health, and animal welfare have driven the expansion of plant-based foods worldwide [1]. Alongside these shifts in consumer preferences, the global market for plant-based foods has experienced rapid growth. Market analyses estimate that the global plant-based food sector may exceed US$100 billion within the next decade, with annual growth rates substantially higher than those observed in the conventional packaged food sector [2].

Plant-based dietary patterns have been associated with several health and environmental benefits, including higher intakes of dietary fiber, polyunsaturated fatty acids (PUFAs), folate, vitamins C and E, and magnesium, as well as lower environmental impacts compared with diets rich in animal-source foods [3,4]. Nevertheless, the adoption of plant-based diets may also present nutritional and practical challenges. Traditional plant protein sources, such as legumes, often require prolonged cooking times, which may limit their convenience for consumers seeking quick and practical meal options [5]. Furthermore, plant foods naturally contain antinutritional compounds, including phytates, tannins, and protease inhibitors, which can impair nutrient bioavailability [6]. Depending on food choices and dietary planning, plant-based diets may also provide insufficient amounts of certain nutrients, such as vitamin B12, iron, zinc, and some indispensable amino acids due to the lower protein quality of individual plant sources [7].

In this context, plant-based meat analogues (PBMAs) have gained significant attention in the global food market as an innovation designed not only to improve convenience and consumer acceptance but also to address some of the nutritional limitations associated with plant-based diets. These products are designed to mimic the sensory, technological, and nutritional characteristics of conventional meat, offering consumers alternatives that resemble traditional meat products such as burgers, sausages, meatballs, and other processed meat derivatives [8].

PBMAs rely on food processing to enhance nutritional quality and sensory acceptance [9]. The development of PBMAs involves multiple technological steps—such as protein isolation, extrusion, structuring, and the incorporation of functional ingredients—which are essential to reproduce the texture, flavor, and overall sensory properties of meat and play a central role in consumer acceptance [10]. In this context, processing should not be interpreted solely as an indicator of reduced nutritional quality, but also as a technological strategy that enables the functionality and acceptability of these products. Consequently, evaluating PBMAs requires a tailored approach that more accurately reflects their nutritional quality.

Given the increasing availability and consumption of PBMAs, there is a growing need for tools capable of assessing their nutritional quality while considering the specific characteristics of these products [11]. In this regard, Nutrition Classification Schemes (NCS) have been widely developed and applied to evaluate the nutritional quality of foods, meals, and overall dietary patterns, supporting public health recommendations, consumer guidance, and nutrition policies. These schemes use predefined criteria or algorithms to classify foods, meals, or dietary patterns according to their nutritional composition and potential health impacts [12].

Among food-based NCS, Nutri-Score is a front-of-pack nutrition labeling system developed in France to facilitate healthier food choices. The system evaluates products according to their content of energy, sugars, saturated fat, sodium, fruits and vegetables, fiber, and protein per 100 g, generating a score that ranges from −15 (healthiest nutritional profile) to +40 (least healthy nutritional profile). This score is subsequently translated into five color-coded categories, from A (dark green) to E (dark orange/red), to facilitate consumer understanding [13].

Similarly, among meal-based NCS, the IQ COSAN (Índice de Qualidade da Coordenação de Segurança Alimentar e Nutricional)—Quality Index of the Food and Nutritional Security Coordination Office—was developed in Brazil to assess the quality of menus within the Brazilian National School Feeding Program (PNAE). Recommended by the National Fund for Educational Development (FNDE), the instrument aims to standardize the qualitative assessment of school menus and verify their compliance with the program guidelines and the principles of adequate and healthy eating established by the Brazilian Dietary Guidelines [14]. The tool evaluates menus based on daily, weekly, and monthly criteria, considering the presence of in natura and minimally processed foods, the limitation of processed and ultra-processed foods, menu diversity, and the inclusion of regional and sociobiodiversity foods. Scores range from 0 to 95 points, classifying menus as “Inadequate”, “Needs Improvement”, or “Adequate”, and providing a standardized approach to support menu planning and improvements in school food quality [14,15].

Finally, the VEGANScreener was recently developed in Europe as a rapid tool to evaluate overall diet quality and nutritional adequacy. The questionnaire comprises 29 items, including questions on the frequency and quality of consumption of key food groups as well as the use of supplements commonly recommended for vegans, such as vitamin B12, vitamin D, iodine, and selenium. Its scoring system combines information on food intake and supplementation, rewarding dietary practices associated with health and sustainability while limiting foods rich in saturated fat, sugar, and sodium. The resulting score provides a comprehensive assessment of nutritional adequacy in vegan dietary patterns, helping to identify potential nutritional gaps and areas for improvement in dietary habits [16].

Although these systems provide valuable approaches for evaluating nutritional quality, they were not specifically designed to assess plant-based meat analogues. Nutri-Score focuses primarily on selected nutrients, while IQ COSAN was developed for menu-level assessment in school settings. Therefore, their direct application to PBMAs may not fully capture the nutritional and technological characteristics that distinguish these products, highlighting the need for tailored assessment tools. In this regard, the development of tailored assessment tools may contribute to a more comprehensive evaluation of PBMAs and support evidence-based decision-making in professional practice. Such tools can assist nutritionists, researchers, and other health professionals in interpreting nutritional information, identifying variability among products, and supporting more consistent nutritional assessment and dietary guidance [17].

Consequently, the assessment of PBMAs requires parameters capable of capturing both nutrients to limit and attributes that should be encouraged in these products. For negative components, sugar, sodium, and saturated and trans fats are widely considered nutrients of concern in public health guidance [18,19]. For PBMAs, it is also essential to ensure nutritional adequacy for critical nutrients when benchmarked against Dietary Reference Intakes (DRI), particularly vitamin B12, iron, and zinc, to reduce the risk of inadequacy in plant-based diets without fortification or supplementation [20,21]. Therefore, beyond sensory characteristics, nutritional evaluation of PBMAs should include protein quality and amino acid adequacy, the presence of polyunsaturated fats, and the use of fortification to support meeting DRI targets [21].

It is also worth noting that, beyond the challenges related to nutritional and sensory quality, this sector remains partially regulated, and specific standards for PBMAs have yet to be established. Regulatory discussions are ongoing worldwide, particularly regarding the naming and designation of these products; however, no consensus has yet been reached. Moreover, these discussions have largely focused on product designation and labeling rather than on establishing specific nutritional standards for PBMAs [22,23].

Therefore, developing practical tools that facilitate the evaluation of PBMAs may help bridge the gap between scientific knowledge and professional practice and support the development of regulatory standards, supporting more informed nutritional guidance while acknowledging the specificities of plant-based meat analogues. Building on previous work, which characterized the nutritional quality of Brazilian PBMAs using existing NCSs [11] and subsequently established PBMA-specific nutritional recommendations through a Delphi consensus [24], the present study aimed to develop a nutrition classification scheme specifically designed for plant-based meat analogues. Specifically, the objectives of this study were (1) to develop a tool to assess the nutritional quality of plant-based meat analogues and (2) to preliminarily assess the tool by applying it to a database of PBMAs and evaluating its perceived usability among nutrition professionals, its intended users.

## 2. Methods

This study was conducted in two phases. The first phase involved the development of a tool designed to assess the nutritional quality of PBMAs. The second phase consisted of testing the proposed tool through its application to a Brazilian database of PBMAs and evaluating its usability through a questionnaire administered to professionals.

### 2.1. Development of the Nutrition Classification Scheme (NCS)–Nutri Veggie Index

The Nutritional Quality Index for Plant-Based Meat Analogues, or NCS–Nutri Veggie Index, was developed based on a consensus established by experts for nutritional recommendations for plant-based meat analogues [24]. In the first round, 10 experts participated in an exploratory online questionnaire to identify key nutritional, sensory, technological, innovative, and regulatory aspects of plant-based meat analogues. Based on their responses and on evidence from the scientific literature, nutritional policies, dietary reference intakes, and nutrient profile models, a comprehensive set of recommendations was developed. In the second round, 20 experts evaluated the recommendations using a 10-point Likert scale for relevance and adequacy. Consensus was defined as an agreement coefficient (AC) of ≥80% for both criteria. Items with an AC below 80% were reformulated and reassessed, whereas items with an AC ≤ 50% were excluded. In the third round, an online workshop was conducted, and 34 participants evaluated the recommendations that had reached consensus in the previous round according to relevance and practical feasibility. Consensus in this round was established when at least 60% of participants assigned a score > 6 for both criteria, with consensus further corroborated when at least two stakeholder groups jointly provided scores > 6.

The Delphi Method was used to reach this consensus, and 12 nutritional recommendations were established: regarding the quality and quantity of proteins (the minimum protein content should be equivalent to traditional meat products, reaching 20% of the recommended daily allowance (RDA) for protein intake per serving or 100 g, prioritizing the use of alternative proteins derived from Brazilian biodiversity species; lipids (the maximum amounts of total and saturated fats should not exceed 30 and 10% of the daily energy value, respectively, and must use ingredients that are sources of lipids with a predominance of mono- and polyunsaturated fatty acids, which in turn provide polyunsaturated fatty acids with an omega 6:omega 3 ratio not exceeding 10:1). Recommendations have also been established for carbohydrates (minimum amount of dietary fiber per serving (20% of the RDA)), for iron and vitamin B12 fortification, and maximum limits for sodium content.

To assist in the development of the NCS–Nutri Veggie Index, a literature search was conducted on other NCS that assess the nutritional quality of foods and menus (Table 1). Thus, Nutri-Score was used as a reference [13], as well as IQ-Cosan [15]. The Brazilian front-of-pack nutrition labeling regulation was used as the reference for the established parameters [25,26].

Based on these references, the proposed NCS–Nutri Veggie Index was structured to combine nutrient-based evaluation criteria with an easy-to-interpret scoring and classification system, enabling a comprehensive assessment of the nutritional quality of PBMA while considering the specific characteristics and recommendations established for these products.

Accordingly, the scoring system assigned negative scores to criteria associated with poorer nutritional quality, such as the absence of protein, protein content below the minimum threshold required for a product to be considered a source of protein, a predominance of saturated fatty acids, excessive saturated fatty acid content, and the presence of front-of-package warning labels. Conversely, positive scores were assigned to attributes associated with improved nutritional quality, including the source and type of protein (protein digestibility, bioavailability, and essential amino acid profile), protein content, total and saturated fat content, the source of unsaturated fatty acids used in the formulation, sodium content, dietary fiber, iron, zinc, vitamin B12, the absence of front-of-package warning labels, and the inclusion of ingredients derived from sociobiodiversity.

The total points assigned to each domain determined its relative contribution to the overall NSC-Nutri Veggie Index score, with the maximum scores across all domains summing to 100 points. Thus, the total points allocated to each domain corresponded to its relative weight (%) in the final NSC score, as detailed in Table 2.

The weighting of the domains was established based on the relative nutritional importance of the different constituents of plant-based meat analogues, considering the fact that these products are intended to mimic conventional meat products and, consequently, are expected to provide relevant amounts of key nutrients associated with meat consumption. In this context, protein content and quality were considered the most important components and were therefore assigned a greater weight in the Index, given the central role of protein in the nutritional value and intended function of these products. Lipid content and quality were assigned the next highest weight, considering both their technological relevance in PBMA formulations and the importance of the fatty acid profile to nutritional quality. The remaining domains were assigned lower weights according to their relative contribution to the overall nutritional profile of these products.

### 2.2. Application of NCS–Nutri Veggie Index Using Brazilian Plant-Based Meat Analogue Database

PBMA products available in the Brazilian market were identified using information collected from food labels originally collected for a previous study conducted by our research group [11]. Data were collected between July 2022 and June 2023 by trained researchers from the Food Labeling Observatory, following a standardized protocol. Data collection was conducted in the five Brazilian regions, including stores from the three largest retail chains in the country, as well as specialized vegetarian/vegan food stores and regional supermarket chains, to capture geographic and product diversity across the Brazilian market. All PBMAs identified at the points of sale that met the eligibility criteria were included in the sample, resulting in a final sample of 334 products.

Eligible products were designed to replace conventional meat products, did not contain ingredients of animal origin, and presented labeling claims such as “vegan”, “vegetarian”, “100% plant-based”, and “veggie”. Labels from products such as burgers, sausages, meatballs, kibbeh, breaded products, ham, bacon, salami, fish, kafta, and other meat analogues were collected [11].

All sides of the packages were photographed using mobile devices to obtain information from the nutrition facts panel, ingredient list, nutritional claims, brand name, package size, and other label characteristics. Product price and GPS location of the point of sale were also recorded. All information was registered in the Brazilian Food Labeling Database using barcode identification to prevent duplicate entries and ensure product traceability.

The NCS–Nutri Veggie Index was subsequently applied to all 334 eligible products included in the final sample. The nutrition facts table and ingredient list were used as the basis for applying the NCS.

The application of the Index to these products was performed by the research team that developed the tool. Initially, one of the researchers independently applied the Nutri-Veggie Index to all products. Subsequently, the other researchers jointly reviewed each product, with particular attention to non-objective aspects, that is, those requiring information from the ingredient list. These aspects included the source and quality of proteins and lipids, omega-3 fortification, and the presence of soluble fiber.

### 2.3. Comparison of the Different Nutritional Classification Schemes

To assess agreement *with* the NCS–Nutri Veggie Index, all PBMAs were classified according to different nutrient classification schemes (NCS): Nutri-Score, Pan American Health Organization, Brazilian front-of-pack labeling thresholds, and NOVA Classification [13,18,25,26,27]. Subsequently, an agreement analysis was performed to assess the consistency between the NCS–Nutri Veggie Index and the selected classification schemes. Table 1 describes all nutritional classification schemes and the criteria used to classify the PBMA.

### 2.4. Questionnaire for NCS–Nutri Veggie Index Usability Evaluation

To evaluate the usability and applicability of the NCS–Nutri Veggie Index, an online questionnaire was developed and distributed to Brazilian nutrition professionals working in the vegan and plant-based food sector. The questionnaire targeted Brazilian nutrition professionals working in or with experience in vegan and plant-based nutrition and/or the plant-based food sector. Participants were recruited using a snowball sampling approach. The questionnaire was based on the USE Questionnaire (Usefulness, Satisfaction, and Ease of Use), developed by Lund [28], which assesses user experience through four dimensions: usefulness, ease of use, ease of learning, and satisfaction. The original instrument contains 30 items scored on a 7-point Likert scale ranging from “strongly disagree” to “strongly agree”. For this study, the questionnaire was translated into Portuguese, and minor adaptations were made to the wording of some items to better fit the context of evaluating a nutritional assessment tool for plant-based meat analogues. Two additional open-ended questions asked participants about the strengths and limitations of the tool. The questionnaire is available in Questionnaire S1.

The questionnaire was widely distributed to Brazilian nutritionists. Participants who agreed to the Free and Informed Consent Form received, by e-mail, a copy of the NCS–Nutri Veggie Index tool, the instruction manual developed to support its use, five photographs of plant-based product labels, and the usability questionnaire. Participants were instructed to apply the tool to at least two of the five labels provided before completing the evaluation questionnaire. Responses were analyzed descriptively according to the mean scores obtained for each USE Questionnaire dimension, aiming to identify strengths, limitations, and potential improvements to the tool.

### 2.5. Statistical Analyses

Results are presented as means (M), standard deviations (SD), medians, and frequencies. To evaluate agreement between the NCS generated by applying the NCS–Nutri Veggie Index and the other four NCS, Cohen’s kappa was used. Agreement was interpreted as follows: 0.01–0.20—slight; 0.21–0.40—fair; 0.41–0.60—moderate; 0.61–0.80—substantial; and 0.81–1.00—excellent.

The internal consistency of the USE Questionnaire was assessed using Cronbach’s alpha. Alpha coefficients were interpreted as follows: <0.50—unacceptable; 0.50–0.59—poor; 0.60–0.69—questionable; 0.70–0.79—acceptable; 0.80–0.89—good; and ≥0.90—excellent.

All analyses were performed using Stata/SE software (version 14.0, Stata Corp, College Station, TX, USA). All figures were generated using the Flourish (2024) tool.

## 3. Results and Discussion

### 3.1. Development of the NCS–Nutri Veggie Index

A nutrition classification scheme was developed called the Nutritional Quality Index for Plant-Based Meat-Analogues (NCS–Nutri Veggie Index). It covers seven areas, with specific scores for each area: 1. Front-of-package nutrition labeling: absence or presence of warning labels, based on the regulatory thresholds established for critical nutrients, including sodium and saturated fat [25]; 2. Proteins: protein content, quality, and source; 3. Lipids: amount of total and saturated fat, source of lipids, and omega-3 fortification; 4. Sodium: amount of sodium; 5. Fiber: amount of fiber and presence or absence of soluble fiber; 6. Micronutrient fortification: presence or absence of iron, zinc, and vitamin B12 fortification; and 7. Sociobiodiversity foods: presence of Brazilian sociobiodiversity foods in the list of ingredients. For each item, positive or negative scores were assigned in accordance with the specific characteristics of each domain. The maximum score is 100 points. Accordingly, based on the NCS–Nutri Veggie Index, PBMAs can be classified as follows: A—Excellent (81–100 points); B—Good (61–80 points); C—Intermediate (41–60 points), D—Low (21–40 points); and E—Poor (0–20 points). The Index is available in Tool S1 as an Excel spreadsheet that can be used to calculate the scores. It is worth emphasizing that the tool uses information provided on food labels. Table 2 summarizes how the scoring system was constructed for each domain. The following section details the process by which the researchers defined the evaluation criteria and assigned the corresponding scores.

Domain 1-Front-of-Pack Nutrition Labeling (FOPNL): Brazil adopts a nutrient profile model based n front-of-pack warning labels for high levels of added sugars, saturated fat, and sodium. Therefore, the cut-off values established in Brazil for sodium (600 mg) and/or saturated fat (6 g) were used as the reference [25]. As PBMAs do not contain significant added sugars in their formulation, this nutrient was not considered in the scoring system. Products without front-of-pack warning labels were assigned a score of 5 points. Conversely, products displaying front-of-pack warning labels received negative scores, with −5 points assigned to products bearing one warning label and −10 points assigned to those bearing two warning labels.

Importantly, these cut-off values can be applied to products marketed worldwide. Several countries have already implemented front-of-pack warning labeling systems, some of which adopt more stringent nutrient thresholds than those established in Brazil. For example, in Colombia, Chile, and Peru, foods containing ≥400 mg of sodium and/or ≥4 g of saturated fat are required to display front-of-pack warning labels [29,30,31].

Domain 2-Protein: Regarding protein, the assessment considered its quantity, quality, and source. It is also noteworthy that the protein domain was assigned a higher weight, given that these foods are expected to serve as meat protein substitutes.

To explain how the index established the protein scoring criteria and why this domain received the highest weight (40 points), it is first necessary to briefly discuss the characteristics of proteins. Understanding the properties of animal proteins is essential for developing plant-based meat analogues (PBMAs) capable of mimicking meat from nutritional, sensory, and textural perspectives.

Meat represents an important source of high-quality protein because it contains all essential amino acids, as well as vitamins and minerals, particularly vitamin B12 and iron. The proteins present in meat are predominantly fibrous proteins, characterized by an elongated structure organized into parallel filaments that form muscle fibers. Approximately 50–55% of the total protein content consists of myofibrillar proteins, primarily myosin and actin, which are responsible for the overall structure and functional properties of muscle. Sarcoplasmic proteins account for approximately 30–34% of the total protein content and are water-soluble. In addition, myoglobin, a globular protein, is responsible for the characteristic red color of meat [32].

In contrast to meat proteins, plant proteins are predominantly globular proteins, characterized by a compact, spherical structure. In general, they denature more readily than fibrous proteins, although their functional properties differ considerably among protein sources. Legumes and oilseeds predominantly contain globulins, whereas pseudocereals are characterized by relatively high levels of albumins. Conversely, cereals mainly contain prolamins and glutelins, except for oats, in which globulins are the predominant storage proteins [33,34,35]. The intrinsic properties of plant proteins necessitate the use of a variety of processing technologies to enable meat substitutes to reproduce desirable sensory attributes, including flavor, juiciness, color, and texture.

Therefore, the index was designed to account for the specific characteristics of these ingredients by comprehensively evaluating their properties and establishing scoring criteria that capture each of these attributes. Protein content varies substantially among plant sources. For example, soybeans contain 30–51% protein, whereas peas and chickpeas contain 24–32% and 19–22% protein, respectively. Therefore, the protein content of a food is one of the key factors determining its suitability as a meat substitute and its ability to achieve a high score in the index. A food is considered a source of protein when it provides at least 10% of the recommended daily intake, and high in protein when it provides at least 20% [25,36,37,38]. Accordingly, products containing at least 20% protein per 100 g received the highest score (15 points), whereas those containing at least 10% protein received 10 points. Products containing between 5% and 10% protein were assigned 5 points. While those containing less than 5% protein received a negative score (−10 points). This scoring system was designed to prioritize products with higher protein contents, given that PBMAs are intended to serve as alternatives to meat products.

However, protein content alone is insufficient to assess the nutritional value of a food, as the nutritional quality of the protein must also be taken into consideration. Accordingly, the Protein Digestibility-Corrected Amino Acid Score (PDCAAS) and the Digestible Indispensable Amino Acid Score (DIAAS) were used as reference criteria to assess protein quality. PDCAAS assesses protein quality by considering both the amino acid composition and protein digestibility. The score reflects the extent to which a protein satisfies human essential amino acid requirements after adjustment for digestibility. In contrast, DIAAS is regarded as the most appropriate method for assessing protein quality, as it evaluates amino acid composition based on the digestibility of individual indispensable amino acids rather than the overall digestibility of crude protein [39,40].

Plant proteins with lower PDCAAS and DIAAS values may contain higher levels of antinutritional factors and may be deficient in specific indispensable amino acids. However, these values can be improved through appropriate processing techniques. Among plant protein sources, soy stands out for its relatively high protein quality, with PDCAAS values ranging from 0.92 to 1.00 and DIAAS values ranging from 95 to 98% [41]. Furthermore, the degree of protein processing also directly affects protein digestibility. For example, soy protein concentrate has a mean DIAAS of 87.7, compared with 85.0 for soy protein isolate and 77.2 for soy flour [42].

The protein content depends on both the source ingredient and the degree of processing. In general, protein ingredients are used in the form of isolates, concentrates, or flours, which contain approximately 90%, 80%, and 50–70% protein, respectively. Thus, the functional properties of proteins are determined by both the extraction process and the food matrix. Furthermore, in addition to protein ingredients, other components, including starch, dietary fiber, lipids, and minerals, can also influence their functional performance in food formulations. For example, non-protein components present in flours, including starch granules, dietary fiber, and lipids, may hinder water penetration, whereas the higher protein content of protein isolates contributes to greater water-holding capacity [43].

Accordingly, products containing protein concentrates or protein isolates as the primary protein source received the highest score (15). Products formulated with textured vegetable protein or protein blends received a score of 10. Whole grains, as well as legume or cereal flours (when no further specification of the protein source was provided), received a score of 5. Products containing no identifiable protein source ingredient received a negative score (−10), such as products based on carrots, broccoli, cabbage, beetroot, and jackfruit.

For protein sources, the Digestible Indispensable Amino Acid Score (DIAAS) was a key criterion in determining the index score. Although most plant proteins contain all indispensable amino acids, they often provide one or more of them in limiting amounts. In general, legumes are limited in sulfur-containing amino acids (methionine and cysteine) and tryptophan, whereas cereals are limited in lysine. Consequently, these protein sources generally exhibit lower DIAAS values [44]. Despite being limited in methionine and cysteine, soy protein has a DIAAS of approximately 100%. Similarly, a combination of soy, potato, and wheat proteins also achieves a DIAAS of 100%, demonstrating the complementary effect of different protein sources. In contrast, other legumes and cereals, when consumed individually, generally exhibit DIAAS values below 70%, including chickpea, lentil, common bean, oat, and wheat proteins. These findings demonstrate that certain protein sources, as well as specific combinations of protein sources, provide superior protein quality [44,45,46].

For the Index, the highest score (10) was assigned to soy protein or combinations of legumes, cereals, and tubers. This was followed by combinations of legumes and cereals or soy and another legume, which received a score of 9. Gluten (8), edamame (7), pea (6), chickpeas or oats or beans or lentils (5), wheat (4). The remaining sources received lower scores: legumes (3), grains (2), low-protein ingredients (1), and very low-protein ingredients (0).

Domain 3-Lipids: Concerning lipids, the assessment considered both the quantity and quality of fat sources, given their relevance.

Fat is a key component of plant-based meat analogues, as it contributes to desirable sensory attributes, including flavor, tenderness, and juiciness. Furthermore, the processing technology used can positively affect the structural characteristics of the final product, such as hardness, springiness, cohesiveness, and gumminess. Therefore, in addition to its sensory and technological functions, the inclusion of fat is essential in PBMA formulations [47].

Although lipids play essential roles in the human body, including maintaining cell membrane structure, supporting metabolism, regulating hormonal signaling, and facilitating the absorption of fat-soluble vitamins, excessive fat intake—particularly of saturated and trans fatty acids—is associated with obesity and an increased risk of chronic non-communicable diseases [19,48].

Accordingly, in line with the World Health Organization (WHO) recommendations to reduce total and saturated fat intake and replace saturated fatty acids with monounsaturated and polyunsaturated fatty acids, the index awarded positive scores to PBMAs containing sources of unsaturated fatty acids in their formulation.

Regarding total fat content, according to the thresholds established by the Pan American Health Organization [18], products in which energy derived from total fat accounted for 30% or more of the total energy (kcal) for serving size received the lowest score (−5 points); as the percentage of energy from total fat decreased, progressively higher scores were assigned. Consequently, products in which energy derived from total fat accounted for less than 6% of the total energy received the highest score (5 points). The same scoring approach was applied to saturated fat, based on the PAHO criterion, which defines 10% of total energy for serving size as the threshold for excessive saturated fat content [18]. Similarly, products in which energy derived from saturated fat accounted for more than 10% of the total energy received the lowest score (−5 points), whereas those in which energy derived from saturated fat contributed less than 2.5% of the total energy for serving size received the highest score (5 points).

In addition to quantity, the quality of lipid sources was considered, as unsaturated fatty acids should be prioritized due to their established cardiometabolic benefits [19]. Products formulated exclusively with unsaturated fatty acids (mono- and polyunsaturated), such as canola or flaxseed, received the highest score (8 points). Products predominantly with unsaturated fatty acids (mono- and polyunsaturated), such as corn, olive, soy, and cottonseed, received 6 points. Products formulated without added fat sources received a score of 0. Conversely, products formulated predominantly with saturated fat sources, such as vegetable fat, palm oil, and coconut oil, received the lowest score (−2).

Beyond the total fat content and the fatty acid profile, fortification with long-chain omega-3 fatty acids was considered an important differentiating factor in PBMAs, given the absence of naturally occurring marine-derived fatty acids in plant-based products. Therefore, the inclusion of eicosapentaenoic acid (EPA) and docosahexaenoic acid (DHA) is essential, as endogenous human synthesis is insufficient to meet physiological requirements. These long-chain omega-3 fatty acids can also be obtained from microalgae-derived sources [49,50]. Furthermore, maintaining an adequate ω-6:ω-3 fatty acid ratio has been associated with a lower risk of non-communicable diseases (NCDs) [51]. Therefore, products fortified with EPA and/or DHA received an additional score (2 points), whereas products without such fortification received no points (0).

Domain 4-Sodium: Sodium is a critical nutrient in PBMAs, as it contributes to flavor enhancement and the masking of undesirable sensory characteristics associated with plant protein ingredients and is therefore frequently present in high concentrations in these products [52]. Given the well-established association between excessive sodium intake and an increased risk of hypertension and cardiovascular diseases [19], the NCS–Nutri Veggie Index adopted the relatively stringent criterion proposed by the PAHO [18], consequently, the higher the sodium content, the lower the score.

Accordingly, products containing ≤1 mg sodium/kcal received the highest score (10 points), whereas products containing 1.1–2 mg sodium/kcal, 2.1–3 mg sodium/kcal, and 3.1–4 mg sodium/kcal received progressively lower scores (5, 2, and 1 point, respectively). Products containing ≥4.1 mg sodium/kcal received no points.

This scoring approach was intended to encourage the development and reformulation of PBMAs with lower sodium contents while maintaining their nutritional quality and sensory acceptability.

Domain 5-Fiber: Regarding dietary fiber, the index considered total fiber content, as PBMAs may contribute to increasing fiber intake compared with conventional meat products, which naturally contain negligible amounts of dietary fiber. Therefore, products providing at least 20% of the DRI for fiber per serving (≥5 g/serving) received the highest score (10 points), whereas products providing 10–19.9% of the DRI (2.5–4.9 g/serving) received 5 points. Products providing 5–9.9% of the DRI (1.3–2.4 g/serving) received no additional points, while products providing less than 5% of the DRI received negative scores, ranging from DRI (0.7–1.2 g/serving) received −2 points and DRI (≤0.6 g/serving) received −5 points according to their fiber content. Negative scores were assigned to products with very low fiber contents to encourage manufacturers to increase the fiber content of PBMAs and enhance their nutritional value.

In addition to total fiber content, the presence of soluble fibers was considered a positive attribute and received an additional score of 2 points. Examples of soluble fiber ingredients included psyllium, flaxseed, apple fiber, pear fiber, oat fiber, β-glucan, and inulin (Soliman, 2019) [53]. Beyond their technological role in improving texture, water retention, and sensory properties in meat analogues, soluble fibers may contribute to intestinal health and promote beneficial modulation of the gut microbiota [54,55,56].

Domain 6-Micronutrient Fortification: Another relevant aspect concerns potential nutritional deficiencies in plant-based diets, due to the absence or low levels of certain micronutrients. Studies evaluating vegan and vegetarian individuals have consistently reported low vitamin B12 intake. Although dietary intakes of iron and zinc appeared to be adequate, the bioavailability of these minerals should be carefully considered, as adequate intake does not necessarily translate into adequate absorption [7].

Therefore, the NCS–Nutri Veggie Index considered fortification with iron, zinc, and vitamin B12 as an important attribute when assessing the nutritional quality of plant-based meat analogues.

Accordingly, products fortified with these micronutrients received positive scores according to the level of fortification, based on the criteria established by Brazilian nutrition labeling regulations and the World Health Organization. Therefore, for a food to be considered a source of vitamins or minerals, it must provide at least 15% of the Reference Daily Value (RDV) of the respective vitamin or mineral per serving. To be considered high in a given vitamin or mineral, the food must provide at least 30% of the respective RDV per serving [25,36]. For iron, products classified as a source (≥2.1 mg per serving) received 2 points, whereas products with high content (≥4.2 mg per serving) received 5 points. For zinc, products classified as a source (≥1.65 mg per serving) received 1 point, and those with high content (≥3.3 mg per serving) received 2 points. Similarly, products classified as a source of vitamin B12 (≥0.36 µg per serving) received 2 points, whereas those with high content (≥0.72 µg per serving) received 5 points. Products without fortification received no additional points.

Although micronutrient fortification remains uncommon among PBMAs currently available on the market, fortification may contribute to improving the nutritional adequacy of plant-based diets and help prevent deficiencies [57].

Domain 7-Sociobiodiversity foods: Finally, the inclusion of ingredients derived from Brazilian sociobiodiversity was considered an important domain in the NCS–Nutri Veggie Index, considering their potential contribution to environmental sustainability, food biodiversity, and the diversification of protein sources used in PBMAs. Accordingly, products containing ingredients derived from Brazilian sociobiodiversity were assigned positive scores (1 point), while products without these ingredients received no additional points (0 points).

Examples of ingredients considered as part of Brazilian sociobiodiversity included species such as baru, babaçu, açaí, cupuaçu, pequi, buriti, jatobá, guaraná, Brazil nuts, and other native or traditionally cultivated species recognized by the Brazilian Sociobiodiversity Food List [58].

It is important to emphasize that the foods included in this domain were selected based on the Brazilian context, where the index was developed. Nevertheless, the underlying principle of this domain is that the selected foods should be adapted to the dietary habits and food availability of the country in which the index is applied. Therefore, the use of native foods that are representative of the local context should be prioritized. A recent FAO report highlights the largely untapped potential of wild foods, identifying more than 10,000 edible species worldwide. Compared with conventional foods, many wild foods exhibit higher nutritional value and may contribute to improving diet quality while supporting biodiversity conservation and fostering more equitable and sustainable food systems [59].

Only the nutritional recommendations established for PBMA through the Delphi method [11] were incorporated into the NCS–Nutri Veggie Index. Therefore, standardizing the nutritional characteristics of these products is essential for establishing identity and quality standards, similar to those already established for animal-derived products [60,61]. In addition, sensory attributes are crucial for the widespread consumer acceptance and consumption of PBMAs.

To assist professionals in using the NCS–Nutri Veggie Index, a manual and an explanatory video were developed and are available as Video S1. The Excel-based tool is also self-explanatory and includes tabs with instructions on the information to be completed (Tool S1). It is also worth highlighting that the manual provides detailed instructions on how the information should be entered into the tool. Therefore, we recommend consulting the manual to ensure the standardized application and use of the tool.

### 3.2. NCS–Nutri Veggie Index Application to the Brazilian PBMA Database

The nutritional classification of 334 PBMAs according to established nutritional classification schemes (NCSs), including Nutri-Score, the Brazilian Nutrient Profile Model, the Pan American Health Organization Nutrient Profile Model, and NOVA, has been previously described using the same database [11] and is described in detail in Table S1. Therefore, in the present study, these classifications were used as reference frameworks to evaluate the performance and agreement of the proposed NCS–Nutri Veggie Index (Table 3). The NCS–Nutri Veggie Index was applied to the same 334 PBMAs, and products were classified according to the five proposed nutritional quality scores (from A to E). Among the products evaluated, 0.6% (*n* = 2) were classified as excellent nutritional quality (A) and 33.5% (*n* = 112) as good quality (B). Most foods had intermediate nutritional quality (C), corresponding to 53.0% (*n* = 177) of the sample. In addition, 6.3% (*n* = 21) of the products were classified as low quality (D) and 6.6% as poor (E) (*n* = 22), showing that about 13% had an unfavorable nutritional profile.

To better understand the nutritional characteristics underlying these classifications, the formulation and nutrient composition of the evaluated PBMAs were examined. Table 4 summarizes the scores assigned to the products across each domain of the index. To facilitate the interpretation of the NCS–Nutri Veggie Index, the domain-specific scores are presented together with a characterization of the products according to their ingredient composition.

Overall, PBMAs showed substantial variability in their formulation, particularly regarding protein sources, protein quality attributes, and lipid composition. Protein content varied considerably across products, ranging from approximately 1 to 54 g/100 g. However, nearly 85% of the products met the criteria to be classified as a source of protein, receiving scores of 15 or 10 points.

Most products (51.8%) were formulated with textured proteins or protein blends, mainly based on combinations of legumes and cereals (35.6%), soy (15.0%), or soy combined with other legumes (12.6%). Concentrated or isolated proteins were present in 12.9% of products, including protein sources such as soy, pea, wheat gluten, and blends of legumes and cereals. A considerable proportion of products (23.1%) relied on whole legumes or cereals as protein sources or did not specify the degree of processing, including ingredients such as chickpeas, lentils, beans, oats, wheat, and edamame. Conversely, 8.1% of products did not contain a relevant protein source, relying primarily on ingredients with low or negligible protein content, such as jackfruit, mushrooms, vegetables, or starch-based ingredients. Nearly half of the products used soy as the predominant protein source, combined with other pulses, including pea, chickpea, and lentil, or pulses with cereals such as oat and wheat.

Lipid composition also differed substantially among products. Most PBMAs (60.8%) contained predominantly unsaturated fatty acid sources, mainly from vegetable oils such as soybean, corn, olive, or cottonseed oils. However, 17.7% of products were characterized by a predominance of saturated fatty acid sources, whereas 21.0% did not contain an added fat source. Only two products (0.6%) relied exclusively on sources of unsaturated fatty acids with more favorable lipid profiles, such as canola or flaxseed oil. Total fat content ranged from 0 to 21.5 g/100 g, while saturated fat ranged from 0 to 17.5 g/100 g. Fortification with long-chain omega-3 fatty acids (EPA/DHA) was uncommon, being identified in only 4 products (1.2%).

The scores assigned for sodium content showed the greatest heterogeneity among all domains. The lower and upper cut-off thresholds were relatively close to each other. This domain underscores the vulnerability of these products in terms of their sodium content. High sodium content has been frequently reported in PBMAs, with some products providing more than half of the recommended daily sodium intake [62,63,64,65,66]. In general, high sodium concentrations are used to improve water retention, enhance texture, and mask residual bitter flavors [67]. However, considering the adverse health effects associated with excessive sodium intake, including increased blood pressure and consequently a higher risk of cardiovascular diseases, reducing sodium levels in PBMA formulations represents one of the major challenges for the development of these products [68].

Regarding dietary fiber, more than 80% of the products met the criteria to be classified as a source of fiber. However, the opposite pattern was observed for ingredients that are sources of soluble fiber, with a considerably lower proportion of products containing these ingredients. Considering the fiber content of PBMAs worldwide, these products can generally be considered sources of dietary fiber and tend to contain higher amounts of fiber than animal-based meat products [69]. However, the type of fiber used has received limited attention in previous studies. Nevertheless, some studies have demonstrated the technological benefits of soluble fibers, particularly in meat analogues, where they can enhance water retention and gel formation, thereby contributing to desirable textural properties and improving the structural stability of the final product [55].

Fortification with iron, zinc, and vitamin B12 was the domain with the lowest scores, indicating that the vast majority of products were not fortified. Micronutrient fortification is not a characteristic unique to products marketed in Brazil, as a similar pattern has been observed in PBMAs marketed in other countries. However, considering that the general population, particularly individuals following vegan diets, may be at risk of micronutrient deficiencies, fortification is highly relevant and may serve as an important public health measure. Furthermore, the chemical form in which these nutrients are provided can directly affect their bioavailability, and this aspect should also be considered when evaluating the nutritional quality of these products [69].

Similarly, ingredients derived from Brazilian sociobiodiversity were rarely included in the formulations. The exploration of new ingredients, particularly locally sourced ingredients from sociobiodiversity, is essential for preserving food systems and represents a strategic pathway for advancing the alternative protein market [70]. Although the inclusion of sociobiodiversity-based ingredients was not common among Brazilian products and, when present, was limited to a single ingredient (cassava), this practice should be encouraged worldwide.

The variability in formulation and nutrient composition was reflected in the distribution of NCS–Nutri Veggie Index scores across products (Figure 1). Only two products, both in the meat-analogue category, achieved category A with a score of 81. However, three additional products from the hamburger, breaded, and meatball categories scored 80, just one point below the threshold for category A. These highest-scoring products were formulated with textured and isolated plant proteins such as pea, rice, and lentils. They were characterized by high protein content (>10 g/100 g) and protein sources and processing characteristics associated with higher protein-domain scores (Table 2), in addition to low sodium levels, favorable lipid profiles characterized by predominantly unsaturated fats, and fortification with iron and vitamin B12. In contrast, the lowest-scoring products were formulated exclusively with fruits or vegetables, particularly jackfruit and cabbage. Consequently, they were characterized by low protein content and limited contribution of other nutrients prioritized by the proposed NCS, such as dietary fiber and favorable lipid sources.

Some products contained predominantly unsaturated fat sources, whereas others were not formulated with added fat sources. Although the absence of added lipids contributed to lower scores in the lipid-quality subdomain, it was not penalized in the proposed index (Table 2). Instead, products simply did not receive the positive score assigned to more favorable lipid sources. This approach recognizes that lipids also play an important technological role in PBMAs. Because plant proteins are predominantly globular and generally exhibit lower emulsifying and structuring capacity than animal proteins [71,72], lipid ingredients are frequently incorporated to improve texture, juiciness, and structural integrity of meat analogues [72,73]. Therefore, these findings do not imply lower nutritional quality of whole plant foods; rather, they highlight that products designed to replace meat should provide nutritional attributes expected from this product category, particularly protein and other nutrients commonly supplied by meat, such as iron and vitamin B12 [74].

Beyond evaluating its ability to discriminate nutritional differences within the PBMA category, the performance of the NCS–Nutri Veggie Index was further examined by assessing its agreement with established nutritional classification schemes (Table 5). It can be observed that the NCS–Nutri Veggie Index showed substantial or excellent agreement with Nutri-Score and the Pan American Health Organization Nutrient Profile Model (PAHO NPM). The agreement with the PAHO NPM was particularly strong for breaded and processed meat analogues, whereas substantial agreement with the Nutri-Score was observed across all product categories. These findings suggest that the NCS–Nutri Veggie Index is consistent with already established nutrition classification schemes. In contrast, fair to moderate agreement was observed with the Brazilian Nutrient Profile Model, while the agreement with NOVA ranged from slight to fair across sales categories.

Discrepancies between nutritional classification schemes are expected because these systems apply different criteria to define nutritional quality, including nutrient composition, food groups, and processing-related characteristics [12,75]. Using the same database of plant-based meat analogues evaluated in the present study, Locatelli et al. [11] also reported differences among NCSs, with nutrient-based approaches showing higher agreement with each other, whereas NOVA exhibited lower agreement with nutrient-based classifications. In the present study, the NCS–Nutri Veggie Index showed substantial to excellent agreement with Nutri-Score and the PAHO NPM, suggesting that the proposed NCS captures nutritional characteristics consistent with established nutrient-based approaches. The strong agreement with the PAHO NPM may be related to this model’s emphasis on nutrients of public health concern and its lower thresholds for critical nutrients compared with other nutrient profile models [12]. These characteristics were also considered in the development of the proposed NCS, as detailed in Table 2.

Conversely, the lower agreement with NOVA reflects the distinct dimensions captured by processing-based and nutrient-based classifications. Most PBMAs are classified as ultra-processed foods because their production typically involves multiple ingredients and processing technologies commonly used to achieve meat-like sensory and functional characteristics [76,77,78]. Consequently, processing-based classifications tend to group products with markedly different nutritional compositions into the same category. Although the degree of processing provides relevant information about food characteristics, processing level alone may not fully capture the nutritional quality of PBMAs, particularly because some technological processes can improve functional properties of ingredients, including protein functionality and, in some cases, bioavailability [79,80].

Taken together, these findings are particularly relevant for consumers and policymakers, as they emphasize that the nutritional evaluation of PBMAs requires consideration of the specific role these products are expected to fulfill within the diet. As PBMAs gain increasing relevance in dietary transitions involving reduced meat consumption [1,66,74], differences in protein content, protein quality-related characteristics, nutrient density, and fortification practices may influence their contribution to dietary intake. The NCS–Nutri Veggie Index provides a framework to capture such differences and may complement existing nutritional classification systems in supporting more informed product evaluation and future regulatory discussions.

### 3.3. NCS–Nutri Veggie Index Usability Test

The usability questionnaire was completed by 11 participants, all of whom were women, with a mean age of 30.64 years (SD = 5.46) and an average of 7.45 years of experience in the field of nutrition (SD = 5.22). Most participants (82%) resided in the state of São Paulo. As shown in Table 6, the most common academic qualification was a postgraduate specialization degree (36%). Nearly all participants worked in clinical practice, and approximately 75% had received training in vegan nutrition. Regarding experience in the vegan market, 36% had worked in the sector for 1–3 years.

The development of the NCS–Nutri Veggie Index was based on a previous Delphi study, in which experts established recommendations regarding nutritional attributes considered relevant for PBMAs [24]. Therefore, the objective of the present study was not to reassess the content validity of the index, but rather to evaluate its usability among professionals representing its intended user group. These approaches address distinct stages of instrument development: while content validation determines whether the selected components adequately represent the intended construct, usability assessment evaluates whether the resulting tool can be understood, interpreted, and applied effectively in practice.

Given the small sample size (*n* = 11), results are presented descriptively. Participants entered between 2 and 5 products into the tool, with five products being the most common number (*n* = 7, 64%). Participants generally reported positive evaluations of the tool across all domains (Table 7). Ease of learning received the highest ratings (M = 6.61, SD = 0.52; median = 6.75), indicating that participants generally considered the tool easy to understand and learn. Usability was also favorably rated (M = 5.96, SD = 0.77; median = 6.00). Ease of use and satisfaction showed similar mean scores (both M = 5.45), with standard deviations of 0.83 and 0.82, respectively. Overall, mean scores ranged from 5.45 to 6.61 on the seven-point scale.

Cronbach’s alpha coefficients ranged from 0.70 to 0.78 across the evaluated domains (usability, α = 0.72; ease of use, α = 0.75; ease of learning, α = 0.78; satisfaction, α = 0.70). These coefficients are reported as descriptive indicators of the internal consistency of the questionnaire and are not interpreted as evidence of the validity of the Index.

Open-ended responses further supported the favorable usability perceptions observed in the quantitative assessment while identifying specific opportunities for refinement. The analysis of the positive comments identified the automatic calculation and final score as the most frequently mentioned strengths (*n* = 4), followed by the clarity of the information and assessment criteria (*n* = 3) and the ease and intuitiveness of the tool (*n* = 3). Participants also highlighted the organized spreadsheet structure and the practical value of having a nutritional score, particularly in clinical practice. Regarding potential improvements, the most frequent comments concerned the clarity and interpretation of the scoring criteria (*n* = 4), particularly the rationale underlying some scores and the classification of protein quality, micronutrient information from the ingredient list, and sociobiodiversity foods. Difficulties related to the assessment of products with multiple protein or lipid sources were reported by three participants. Two participants commented on the organization and navigation of the tool, while one participant reported a technical error in the automatic score calculation, which may have been related to the spreadsheet-based implementation of the tool. One participant also questioned the correspondence between the final score and their perceived healthfulness of a product they considered highly processed, while two participants did not identify any needed improvements.

Regarding intended use, six participants indicated that they would use the tool in their professional practice; one stated that they would recommend it despite it not being directly relevant to their area of practice, and two indicated that they might use it following further improvements. Participants also mentioned potential applications such as supporting nutritional recommendations and building databases of products commonly used by patients. One participant suggested using image recognition and artificial intelligence to automate product data entry, which could potentially facilitate the application of the tool in future versions. These observations indicate that clearer instructions and worked examples could further facilitate the application of the predefined criteria, particularly for products with more complex formulations. Future versions of the tool could also explore AI-assisted data entry, whereby product information could be extracted from labels or ingredient lists to support application of the Index, potentially reducing manual data entry and simplifying its use in professional practice.

The favorable findings observed in this preliminary assessment are relevant because assessing the nutritional quality of plant-based meat analogues requires the integration of multiple nutritional attributes, including nutrient composition, protein characteristics, ingredient sources, and fortification practices. Such multidimensional assessments can be challenging due to the number of methodological decisions required to define and apply nutrient profiling approaches [81]. For example, nutrient profiling models such as the Health Star Rating and Nutri-Score were developed through expert-based processes involving the selection and weighting of nutritional attributes to support food classification and consumer-oriented communication [13,81]. Beyond the technical validity of such approaches, usability and user experience are recognized as important components for the effective implementation of decision-support tools, as they influence whether professionals can understand, apply, and incorporate these systems into practice [82].

Although only 11 nutrition professionals participated in the usability assessment, this sample size is compatible with formative usability testing, in which relatively small samples can identify a substantial proportion of usability problems, particularly during early stages of tool development [83,84]. However, the small sample size and the concentration of participants in the Southeast region of Brazil, particularly in the state of São Paulo, limit the generalizability of the findings. In addition, all participants were women, and most had experience in plant-based nutrition, which may have contributed to the favorable perceptions observed. Therefore, these findings should be interpreted as preliminary evidence of perceived usability among a specialized group of nutrition professionals rather than as a comprehensive evaluation of the Index’s usability across different professional contexts. Further studies with larger and more geographically and professionally diverse samples are needed to assess the reproducibility and usability of the Index across different user groups and settings.

### 3.4. Study Limitations

Several limitations of the present study should be acknowledged. First, the Nutri-Veggie Index was developed and applied to products available on the Brazilian market. Although its criteria are informed by internationally established nutritional recommendations and classification approaches, differences in food composition, labeling regulations, fortification practices, and product formulations may limit its applicability to other countries. Further adaptation and validation in different national and regulatory contexts are therefore warranted.

Second, the Index is based on information declared on food labels, including the Nutrition Facts table and ingredient lists, rather than laboratory measurements. Thus, it may not fully reflect actual nutrient composition or nutrient bioavailability. In particular, the current version does not systematically account for the chemical forms of micronutrients or other factors affecting nutrient bioavailability.

Third, the Index was not validated against clinical, epidemiological, or other health-related outcomes. Therefore, it should be interpreted as a framework for assessing the nutritional quality of PBMAs based on label-declared information, rather than as a predictor of health outcomes. Future studies should evaluate its validity in relation to laboratory-measured nutrient composition and relevant health outcomes.

Fourth, formal inter-rater and intra-rater reliability were not assessed. The Index was initially applied to all 334 products by one researcher and subsequently reviewed jointly by the other members of the research team, particularly for criteria requiring interpretation of ingredient-list information. However, no independent repeated assessment of a subsample was performed. Although joint review and consensus helped standardize the application of the Index, this approach does not replace formal reliability assessment. Future validation studies should include independent raters, repeated scoring of a representative subsample, and appropriate reliability analyses.

Finally, the weighting system was established according to the relative nutritional importance of the different domains for PBMAs, with greater weight assigned to protein quantity and quality, followed by lipid quantity and quality, and lower weights to the remaining domains. Although this approach was informed by nutritional recommendations and the intended nutritional role of these products, the weighting scheme was not empirically derived or evaluated through a formal sensitivity analysis. Therefore, the effect of alternative weighting schemes on the final classification remains uncertain. Future studies should assess the robustness of the Index under alternative weighting scenarios.

## 4. Conclusions

The application of the NCS–Nutri Veggie Index to 334 Brazilian PBMAs demonstrated substantial variability in the nutritional quality of products available on the market, with most products classified as intermediate nutritional quality (category C). The index was able to discriminate differences in formulation and nutrient composition within and across product categories, particularly regarding protein content and quality-related characteristics, lipid profile, sodium content, and micronutrient fortification. Furthermore, the NCS–Nutri Veggie Index showed substantial to excellent agreement with the PAHO Nutrient Profile Model and the Nutri-Score, supporting its consistency with established nutrient-based classification approaches.

The usability assessment indicated favorable perceptions regarding ease of learning, ease of use, usefulness, and satisfaction among the participating nutrition professionals. Nevertheless, these findings should be interpreted cautiously due to the small sample size, and further studies with larger and more diverse samples are needed to confirm usability.

Thus, the findings indicate that the NCS–Nutri Veggie Index may provide a relevant framework for assessing the nutritional quality of PBMAs, a heterogeneous category that includes products with markedly different nutritional profiles and expected dietary roles. By incorporating criteria particularly relevant to meat substitutes, such as protein quantity, protein quality-related characteristics, and fortification with nutrients commonly supplied by meat, the index may complement existing generic nutritional classification systems and may support more informed evaluation by researchers, health professionals, and policymakers.

This research envisions, as a future perspective, the development of an artificial intelligence tool capable of automating label reading for index calculation by nutrition professionals. In addition, as the tool was developed for meat analogues, it is intended to adapt the instrument for dairy analogues, considering their specific characteristics, as well as the importance of assessing their nutritional quality. Although the current version was developed within the Brazilian regulatory and food labeling context, future studies could investigate its adaptation to other countries, considering differences in regulatory and food labeling requirements, followed by appropriate evaluation of its applicability and performance in those contexts.

## Figures and Tables

**Figure 1:**
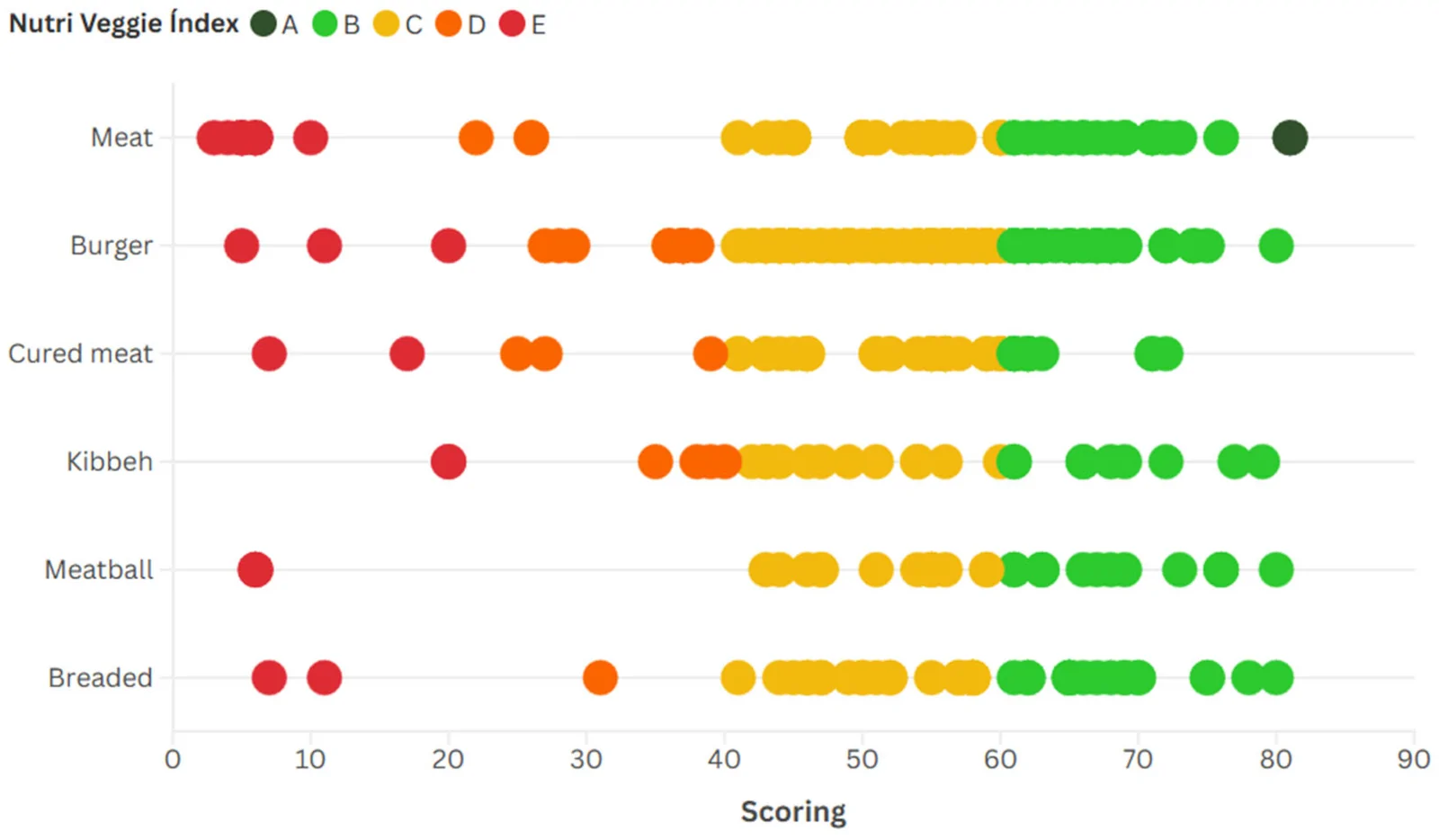
Scoring of the PBMAs using the NCS–Nutri Veggie Index, according to the sales category.

**Table 1:** Nutrition classification schemes and criteria used in this study.

Nutrition Classification Schemes	Nutrients of Concern	Classification Good Nutritional Profile	Classification Poor Nutritional Profile
Nutrient Profiling Models			
NutriScore (France, Ministry of Health and Prevention, 2017)	Algorithm that evaluates the nutritional composition of products (sugars, saturated fat, sodium, fiber, and protein per 100 g/100 mL), classifying them from A (best nutritional quality) to E (worst nutritional quality).	A–C	D–E
Pan American Health Organization (Pan American Health Organization, 2016)	Sugar (free sugars): ≥10% of total energy Sodium: ≥1 mg per 1 kcal Saturated fat: ≥10% of total energy Trans fat: ≥1% of total energy Presence of nonnutritive sweeteners	No warning label	Have at least one warning label
Brazil FOPL (Brazil, National Health Surveillance Agency, 2020)	Added sugar: ≥15 g/100 g; ≥7.5 g/100 mL Sodium: ≥600 mg/100 g; ≥300 mg/100 mL Saturated fat: ≥6 g/100 g; ≥3 g/100 mL	No warning label	Have at least one warning label
Food-Based Profiling Model			
NOVA Classification (Brazil, Ministry of Health, 2014)	Unprocessed or minimally processed foods are natural or slightly altered foods without added ingredients. Processed culinary ingredients are substances extracted from foods, such as oils or sugar. Processed foods combine natural foods with added salt, sugar, or fat. Ultra-processed foods are industrial formulations containing food derivatives and additives.	In natura, minimally processed, and processed food	Ultra-processed food

**Table 2:** Attribution of points by domain.

Assessment		Item	Score	Total Points
1. Front-of-Package Nutrition Labeling		Absence of warning labels	5	5
		Presence of 1 warning label	−5	
		Presence of 2 warning labels	−10	
2. Protein	Quantity	≥20% DRI/100 g (≥10 g/100 g)	15	40
		10–19.9% DRI/100 g (5–10 g/100 g)	10	
		5–9.9% DRI/100 g (2.5–5 g/100 g)	5	
		<4.9% DRI/100 g (<2.5 g)	−10	
	Quality	Concentrated or isolated	15	
		Protein from...; Textured; Protein blend	10	
		Whole grain; when there is no specification; legume or cereal flour	5	
		No protein source.	−10	
	Source	Soy; legume + cereal + tuber	10	
		Pulse + cereal; Soy + other pulse	9	
		Gluten	8	
		Edamame	7	
		Pea	6	
		Chickpeas; oats; beans; lentils	5	
		Wheat	4	
		Other sources of legumes	3	
		Other sources of grains	2	
		Other low-protein ingredients (mushrooms)	1	
		Other very low-protein ingredients (jackfruit)	0	
3. Lipids	Total fat	≤5.9% DRI for energy (≤118 kcal/serving size)	5	20
		6–13.9% DRI for energy (119–279 kcal/serving size)	2	
		14–21.9% DRI for energy (280–439 kcal/serving size)	0	
		22–29.9% DRI for energy (400–599 kcal/serving size)	−2	
		≥30% DRI for energy (600 kcal/serving size)	−5	
	Saturated fat	≤2.4% DRI for energy (≤48 kcal/serving size)	5	
		4.9–2.5% DRI for energy (49–98 kcal/serving size)	2	
		7.4–5% DRI for energy (99–148 kcal/serving size)	0	
		7.5- 9.9% DRI for energy (149–198 kcal/serving size)	−2	
		≥10% DRI for energy (≥199 kcal/serving size)	−5	
	Quality	Exclusive use of unsaturated fatty acid sources (mono- and polyunsaturated), such as canola or flaxseed	8	
		Predominance of unsaturated fatty acids (mono- and polyunsaturated), such as corn, olive, soy, and cottonseed	6	
		Absence of fatty acids	0	
		Predominance of saturated fatty acids (vegetable fat, palm oil, coconut oil)	−2	
		Presence of omega-3 fortification (EPA ** and DHA ***).	2	
		Absence of omega-3 fortification	0	
4. Sodium		≤1 mg/kcal	10	10
		1.1–2 mg/kcal	5	
		2.1–3 mg/kcal	2	
		3.1–4 mg/kcal	1	
		≥4.1 mg/kcal	0	
5. Fibers	Source	≥20% DRI (≥5 g/serving size)	10	12
		10–19.9% DRI (2.5–4.9 g/serving size)	5	
		5–9.9% DRI (2.4–1.3 g/serving size)	0	
		2.5–4.9% DRI (0.7–1.2 g/serving size)	−2	
		≤2.4% DRI (≤0.6 g/serving size)	−5	
	Soluble fibers	Presence of soluble fibers	2	
		Absence of soluble fibers	0	
6. Fortification	Iron	High content (≥4.2 mg/serving size)	5	12
		Source (≥2.1 mg/serving size)	2	
		Not fortified	0	
		High content (≥3.3 mg/serving size)	2	
	Zinc	Source (≥1.65 mg/serving size)	1	
		Not fortified	0	
	Vitamin B12	High content (≥0.72 mg/serving size)	5	
		Source (≥0.36 mg/serving size)	2	
		Not fortified	0	
7. Sociobiodiversity foods		Presence of sociobiodiversity foods.	1	1
		Absence of sociobiodiversity foods.	0	

DRI: Daily recommended intake; ** EPA: eicosapentaenoic acid; *** DHA: docosahexaenoic acid. Energy reference values: 2000 kcal; DRI Protein: 50 g; DRI Fiber: 25 g fiber; DRI Iron: 14 mg; DRI Zinc: 11 mg; DRI Vitamin B12: 2.4 mg.

**Table 3:** Frequency of classification of plant-based meat analogues, according to NCS–Nutri Veggie Index, grouped according to sales category.

Nutri Veggie Index	Meatball *n* (%)	Meat *n* (%)	Cured Meat *n* (%)	Breaded *n* (%)	Burger *n* (%)	Kibbeh *n* (%)
A	0 (0.0)	2 (2.9)	0 (0.0)	0 (0.0)	0 (0.0)	0 (0.0)
B	14 (46.7)	22 (32.4)	12 (25.0)	18 (40.9)	37 (31.6)	9 (33.3)
C	13 (43.3)	31 (45.6)	31 (64.6)	23 (52.3)	67 (57.3)	12 (44.5)
D	0 (0.0)	3 (4.4)	3 (6.2)	1 (2.3)	10 (8.5)	4 (14.8)
E	3 (10.0)	10 (14.7)	2 (4.2)	2 (4.5)	3 (2.6)	2 (7.4)
Total	30 (100.0)	68 (100.0)	48 (100.0)	44 (100.0)	117 (100.0)	27 (100.0)

**Table 4:** Scoring of the PBMAs using the NCS–Nutri Veggie Index, according to each domain.

Assessment Item		Score	*n*	%
1. Front-of-Package Nutrition Labeling		5	235	70.4
		−5	92	27.5
		−10	7	2.1
2. Proteins	Quantity	15	223	66.8
		10	60	18.0
		5	34	10.2
		−10	17	5.1
	Quality	15	43	12.9
		10	173	51.8
		5	91	27.2
		−10	27	8.1
	Source	10	67	20.1
		9	161	48.2
		8	3	0.9
		7	1	0.3
		6	19	5.7
		5	32	9.6
		4	11	3.3
		3	0	0
		2	15	4.5
		1	1	0.3
		0	24	7.2
3. Lipids	Total Fat	5	288	86.2
		2	45	13.5
		0	0	0.0
		−2	0	0.0
		−5	1	0.3
	Saturated Fat	5	295	88.3
		2	34	10.2
		0	4	1.2
		−2	1	0.3
		−5	0	0.0
	Quality	8	2	0.6
		6	203	60.8
		0	70	21.0
		−2	59	17.7
		0	330	98.8
		2	4	1.2
4. Sodium		10	49	13.5
		5	82	24.6
		2	104	31.1
		1	54	16.2
		0	45	13.5
5. Fibers	Source	10	160	47.9
		5	118	35.3
		0	33	9.9
		−2	9	2.7
		−5	14	4.2
	Soluble fiber	2	59	17.7
		0	275	82.3
6. Fortification	Iron	5	15	4.5
		2	43	12.9
		0	276	82.6
	Zinc	2	1	0.3
		1	7	2.1
		0	326	97.6
	Vitamin B12	5	37	11.1
		2	19	5.7
		0	278	83.2
7. Sociobiodiversity foods		1	100	29.9
		0	234	70.1

**Table 5:** Agreement between the Nutri Veggie Index and each nutritional classification scheme measured by Cohen’s κ coefficient (with a 95% Confidence Interval) for plant-based meat analogues.

	Nutri Veggie Index/Nutri-Score	Nutri Veggie Index/NPM PAHO	Nutri Veggie Index/NPM Brazil	Nutri Veggie Index/NOVA
Category	K CI (min; max)	K CI (min; max)	K CI (min; max)	K CI (min; max)
Burger	0.70 * (0.62; 0.79)	0.77 * (0.70; 0.85)	0.32 (0.23; 0.41)	0.34 (0.23; 0.41)
Breaded	0.79 * (0.67; 0.91)	0.81 ** (0.69; 0.93)	0.20 (0.08; 0.32)	0.25 (0.11; 0.38)
Kibbeh	0.77 * (0.61; 0.94)	0.70 * (0.51; 0.88)	0.33 (0.14; 0.52)	0.37 (0.17; 0.56)
Cured meat	0.70 * (0.57; 0.84)	0.89 ** (0.80; 0.98)	0.45 (0.31; 0.60)	0.04 (−0.01; 0.10)
Meatball	0.80 * (0.64; 0.95)	0.73 * (0.56; 0.90)	0.36 (0.18; 0.54)	0.20 (0.04; 0.35)
Meat	0.72 * (0.61; 0.83)	0.80 * (0.71; 0.90)	0.45 (0.33; 0.57)	0.10 (0.02; 0.17)

* Substantial agreement; ** excellent agreement.

**Table 6:** Descriptive characteristics of participants.

Characteristic	*n*	% of Total
Brazilian region of residence		
Southeast (Espírito Santo and São Paulo)	10	90%
South (Santa Catarina)	1	10%
Highest level of qualification		
Undergraduate	1	9%
Postgraduate specialization	4	36%
Master’s degree	2	18%
Doctoral degree	3	27%
Postdoctoral training	4	36%
Professional activity		
Clinical practice	4	36%
Academic research or teaching	4	36%
Non-governmental organization focused on plant-based diets	2	18%
Food service	1	9%
Training in plant-based nutrition		
Postgraduate specialization	2	18%
Master’s or doctoral degree	1	9%
Complementary training (courses, extension programs, and workshops)	5	45%
No specific training in the field	3	27%
Years of experience in the plant-based food sector		
Less than 1 year	1	9%
1–3 years	4	36%
4–6 years	3	27%
≥7 years	3	27%

**Table 7:** Descriptive statistics for usability, ease of use, ease of learning, and satisfaction.

	Usability	Ease of Use	Ease of Learning	Satisfaction
Mean (SD) *	5.96 (0.77)	5.45 (0.83)	6.61 (0.52)	5.45 (0.82)
Median *	6.00	5.50	6.75	5.40
Min–max *	4.57–6.86	4.30–6.80	5.50–7.00	4.00–6.80

Note. * Responses were collected on a 7-point Likert scale.

## Data Availability

The original contributions presented in this study are included in the article/Supplementary Materials. Further inquiries can be directed to the corresponding author.

## Supplementary Materials

The following supporting information can be downloaded at: https://www.mdpi.com/article/10.3390/foods15193416/s1, Questionnaire S1: Questionnaire to evaluate the usability and applicability of Nutri Veggie Index; Video S1: Video tutorial and user manual of Nutri Veggie Index; Tool S1: Tool CNS–Nutri Veggie Index; Table S1: Frequency of classification of plant-based meat analogues, according to each nutrition classification scheme, grouped according to sales category. Figure S1: Classification of PBMAs using the NCS–Nutri Veggie Index, according to the sales category. Note. “Meatba …” = meatball.

## Abbreviations

The following abbreviations are used in this manuscript:

DHA	Docosahexaenoic Acid
DIAAS	Digestible Indispensable Amino Acid Score
DRI	Dietary Reference Intake
EPA	Eicosapentaenoic Acid
FNDE	National Fund for Educational Development
NCDs	Non-Communicable Diseases
NCS	Nutrition Classification Scheme
NPM	Nutrient Profile Model
PAHO	Pan American Health Organization
PBMA	Plant-Based Meat Analogues
PDCAAS	Protein Digestibility-Corrected Amino Acid Score
PNAE	Brazilian National School Feeding Program
PUFAs	Polyunsaturated Fatty Acids
RDA	Recommended Daily Allowance
