# Supplementary material for: Nutri Veggie Index: An Innovative Nutrition Classification Scheme for Quality Assessment of Plant-Based Meat Analogues
Source: Foods. 2026 Sep 24;15(19):3416. doi: 10.3390/foods15193416 (PMC13664401; doi:10.3390/foods15193416)
Supplement: Supplementary file 1 [file foods-15-03416-s001.zip › foods-4543005-supplementary.pdf]

Nutri Veggie Index: An Innovative Nutrition Classification Scheme for Quality Assessment of Plant-Based Meat Analogues

Supplementary Materials

Questionnaire S1. Questionnaire to evaluate the usability and applicability of Nutri Veggie Index.

**Domain**	**Question**	**Response**
Sociodemographic data	What is your age in completed years?	Whole numbers only
	What is your gender?	Multiple choice:<br>● Woman<br>● Man<br>● Non-binary<br>● Prefer not to answer<br>● Other, please specify:
	In which Brazilian state do you currently live?	Multiple choice:<br>● List of the 26 Brazilian states and the Federal District
	What is your highest level of education?	Multiple choice:<br>● Undergraduate degree<br>● Lato sensu graduate degree (specialization)<br>● Master's degree<br>● PhD<br>● Postdoctoral degree
Professional profile	What is your professional field of work in the vegan/plant-based sector?	Check all that apply:<br>● Clinical practice<br>● Product development or formulation (industry)<br>● Communications and marketing<br>● Academic research or teaching<br>● Food services<br>● Retail/business/entrepreneurship (e.g., restaurants, brands, services)<br>● Consulting or technical advisory<br>● Other, please specify:
	Do you have specific training to work in the vegan/plant-based food sector?	Multiple choice:<br>● Additional training (courses, extension programs, professional training, workshops)<br>● Specialization (lato sensu postgraduate degree)<br>● Academic training

		(master's/doctoral degree)<br>• I do not have specific training in this area
	How many years have you worked in the vegan/plant-based market?	Multiple choice:<br>• Less than 1 year<br>• 1–3 years<br>• 4–6 years<br>• 7–10 years<br>• More than 10 years
Product entry check	How many plant-based meat analogue products did you enter into the tool?	Multiple choice:<br>• 2 products<br>• 3 products<br>• 4 products<br>• 5 products<br>• More than 5 products
Utility	The tool helped me be more effective in assessing the nutritional quality of plant-based meat analogue products.	7-point Likert scale: Totally disagree – Totally agree
	The tool helped me assess the nutritional quality of plant-based meat analogue products more productively.	
	The tool can give me greater control over the nutritional analyses I perform.	
	It can make the analyses I want to perform easier to complete.	
	It saves me time when I use it.	
	It meets my needs.	
	It does everything I would expect it to do.	
Ease of use	It is easy to use.	7-point Likert scale: Totally disagree – Totally agree
	It is simple to use.	

	It is user-friendly.	
	It requires the fewest possible steps to accomplish what I want to do with it.	
	Using it does not require much effort.	
	I can use it without written instructions.	
	I did not notice any inconsistencies while using it.	
	Both occasional and regular users would like it.	
	I can quickly and easily correct errors.	
	I can use it successfully every time.	
Ease of learning	I learned to use it quickly.	7-point Likert scale: Totally disagree – Totally agree
	I can easily remember how to use it.	
	I became skilled at using it quickly.	
	It is easy to learn how to use it.	
Satisfaction	I am satisfied with the tool.	7-point Likert scale: Totally disagree – Totally agree
	I would recommend it to a colleague.	

	It works the way I want it to work.	
	I feel that I need it.	
	It is pleasant to use.	
Overall perceptions and intended use	What did you like most about the tool?	Open-ended question
	Is there anything that could be improved? If so, what?	
	Would you use it in your professional practice?	

Note: The questionnaire was administered in Brazilian Portuguese.

Video S1: Video tutorial and user manual for the Nutri Veggie Index (Video manual, https://accounts.google.com/v3/signin/identifier?continue=https://drive.google.com/drive/folders/1sO2Ghry0D4QbxGHjJ8E-woRUCS3-8-tP&followup=https://drive.google.com/drive/folders/1sO2Ghry0D4QbxGHjJ8E-woRUCS3-8-tP&osid=1&passive=1209600&service=wise&flowName=GlifWebSignIn&flowEntry=ServiceLogin&dsh=S1041305551:1790233136169846)

Table S1. Frequency of classification of plant-based meat analogues, according to each nutrition classification scheme, grouped according to sales category.

	**Meatball n (%)**	**Meat n (%)**	**Cured meat n (%)**	**Breaded n (%)**	**Burger n (%)**	**Kibbeh n (%)**
**Nutri-Score**						
A	11 (36.7)	22 (32.3)	13 (27.1)	13 (36.4)	46 (39.3)	8 (29.6)
B	4 (13.3)	15 (22.1)	13 (27.1)	9 (20.5)	17 (15.5)	8 (29.6)
C	12 (40.0)	23 (33.8)	9 (18.8)	11 (25.0)	25 (21.4)	11 (40.7)
D	2 (6.7)	7 (10.3)	11 (22.9)	8 (18.2)	27 (23.1)	0 (0.0)
E	1 (3.3)	1 (1.5)	2 (4.2)	0 (0.0)	2 (1.7)	0 (0.0)
**NPM Brazil**						

High in sodium						
Yes	6 (20.0)	21 (30.9)	20 (41.7)	6 (13.6)	20 (17.1)	2 (7.4)
No	24 (80.0)	47 (69.1)	28 (58.3)	38 (86.4)	97 (82.9)	25 (92.6)
High in saturated fat						
Yes	2 (6.7)	3 (4.4)	3 (6.3)	2 (4.6)	20 (17.1)	1 (3.7)
No	28 (93.3)	65 (95.6)	45 (93.8)	42 (95.5)	97 (82.9)	26 (96.3)
High in sugar						
Yes	0 (0.0)	0 (0.0)	0 (0.0)	0 (0.0)	0 (0.0)	0 (0.0)
No	30 (100.0)	68 (100.0)	48 (100.0)	44 (100.0)	117 (100.0)	27 (100.0)
**NPM PAHO**						
Sodium						
Yes	25 (83.3)	58 (85.3)	43 (89.6)	37 (84.1)	98 (83.8)	25 (82.6)
No	5 (16.7)	10 (14.7)	5 (10.4)	7 (15.9)	19 (16.2)	2 (7.4)
Total fats						
Yes	0 (0.0)	0 (0.0)	1 (2.1)	0 (0.0)	0 (0.0)	0 (0.0)
No	30 (100.0)	68 (100.0)	47 (97.9)	44 (100.0)	117 (100.0)	27 (100.0)
Saturated fats						
Yes	0 (0.0)	0 (0.0)	0 (0.0)	0 (0.0)	0 (0.0)	0 (0.0)
No	30 (100.0)	68 (100.0)	48 (100.0)	44 (100.0)	117 (100.0)	27 (100.0)
Trans fats						
Yes	0 (0.0)	0 (0.0)	0 (0.0)	0 (0.0)	0 (0.0)	0 (0.0)
No	30 (100.0)	68 (100.0)	48 (100.0)	44 (100.0)	117 (100.0)	27 (100.0)
Sugar						
Yes	0 (0.0)	0 (0.0)	0 (0.0)	0 (0.0)	0 (0.0)	0 (0.0)
No	30 (100.0)	68 (100.0)	48 (100.0)	44 (100.0)	117 (100.0)	27 (100.0)
Artificial sweeteners						
Yes	0 (0.0)	0 (0.0)	4 (8.3)	0 (0.0)	0 (0.0)	0 (0.0)

No	30 (100.0)	68 (100.0)	44 (91.7)	44 (100.0)	117 (100.0)	27 (100.0)
**NOVA**						
In natura and minimally processed food	0 (0.0)	6 (8.8)	0 (0.0)	0 (0.0)	0 (0.0)	0 (0.0)
Processed food	9 (30.0)	8 (11.8)	3 (6.3)	14 (31.8)	37 (31.6)	14 (51.9)
Ultra-processed food	21 (70.0)	54 (79.4)	45 (93.8)	30 (68.2)	80 (68.4)	13 (48.2)
**Total**	**30 (100.0)**	**68 (100.0)**	**48 (100.0)**	**44 (100.0)**	**117 (100.0)**	**27 (100.0)**

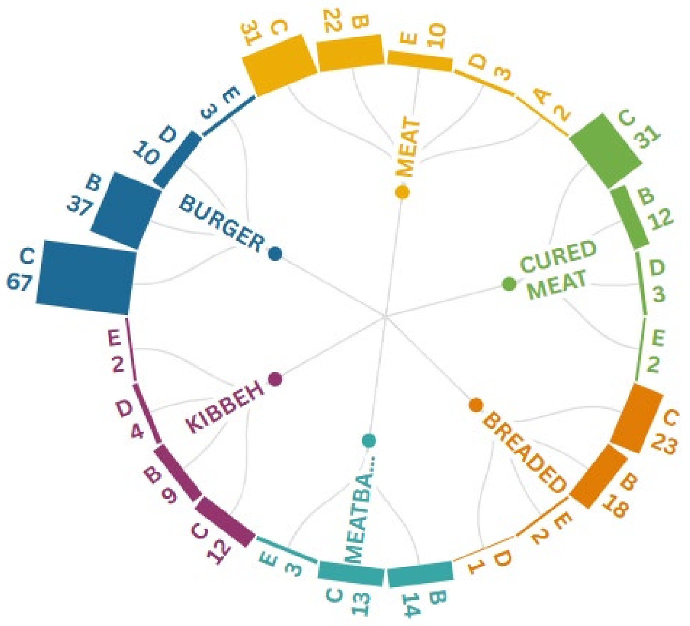

Figure S1. Classification of the PBMAs using the NCS-Nutri Veggie Index, according to the sales category. *Note.* "Meatba…" = meatball.

Tool S1. Tool CNS–Nutri Veggie Index (Nutri Veggie Index.english.xlsx - Google Planilhas)
